# Umbilical cord blood collection at birth reduces early iatrogenic blood loss in preterm infants with birth weights <1,500 g

**DOI:** 10.3389/fped.2026.1739158

**Published:** 2026-03-12

**Authors:** Huihui Wu, Lin Cheng, Jiajun Lei, Jiaolin Chu, Tao Bo

**Affiliations:** 1 Neonatal Intensive Care Unit, Department of Pediatrics, The Third Xiangya Hospital of Central South University, Changsha, Hunan, China; 2 Hunan University of Traditional Chinese Medicine, Zhuzhou, Hunan, China

**Keywords:** blood transfusion, neonatal anemia, premature infants, umbilical cord blood, very low birth weight infants

## Abstract

**Background:**

Frequent blood collection leads to anemia and increased transfusion needs. Umbilical cord blood (UCB) collection has been suggested to reduce the need for subsequent blood draws, but its effectiveness, especially in very low birth weight infants (VLBWI), remains insufficiently explored.

**Methods:**

This retrospective study was approved by the Ethics Committee of Xiangya Third Hospital, Central South University (*N*o. 23673), which granted a waiver of informed consent in accordance with national regulations and institutional guidelines, stratifying infants into two groups based on whether UCB was collected at birth: the UCB collection (UCB, 68 infants) group and the non-umbilical cord blood collection (NUCB, 70 infants) group. These cohorts were further analyzed according to birth weight (BW), with 64 infants with BW ≥1,250 g (UCB=21, NUCB=43) and 74 infants with BW < 1,250 g (UCB=47, NUCB=27). Clinical data were then collected and analyzed.

**Results:**

Among preterm infants with BW < 1,250 g, the occurrence of hemoglobin (Hb) < 120 g/L within the first week after birth, the volume of blood drawn within the first 24 h and the first three days after birth were significantly lower in the UCB group than in the NUCB group (*P* < 0.05); the rate of non-transfusion within 7 days after birth was higher in the UCB group than that in the NUCB group (*P* < 0.05). In preterm infants with BW ≥1,250 g, the volume of blood drawn within 24 h and within the first three days after birth was significantly lower in the UCB group than in the NUCB group (*P* < 0.05).

**Conclusion:**

UCB can effectively reduce the volume of blood drawn from VLBWI in the early postnatal period, and help protect those preterm infants from adverse stimuli.

**Trial registration:**

Chinese Clinical Trial Registry, ChiCTR2500113741.

## 1 Introduction

With the rapid advancement of neonatal intensive care unit (NICU), the survival rate of premature infants especially extremely premature infants has significantly improved in the past thirty years (1). However, as BW decreases, the associated risk of systemic complications and mortality also increases (2). The infants' conditions are unstable. Blood collection is required for laboratory testing. However, very low birth weight infant (VLBWI), whose BW <1,500 g, is at the risk of hemodynamic fluctuations during blood drawn due to their small size and low blood volume, and repeated blood drawn can lead to iatrogenic anemia. Moreover, the pain of blood drawing may induce intracranial hemorrhage by increasing blood pressure in premature infants (3, 4). Some studies have suggested that greater the amount of blood drawn, the higher risk of iatrogenic anemia, which is detrimental to clinical outcomes in premature infants (5, 6). The frequent need for blood drawn in VLBWI has been associated with poorer clinical outcomes, including a higher incidence of blood transfusions and an elevated risk of severe anemia, both of which may contribute to long-term developmental complications (7). This underscores the necessity for innovative strategies aimed at minimizing the detrimental effects of blood sampling in this vulnerable population. After the umbilical cord is clamped, A large amount of fetal blood is still stored in the umbilical cord and the placenta, which is often discarded. It is unclear whether collecting this fetal blood can be used for early laboratory tests in premature infants, thereby reducing iatrogenic blood loss and ensuring sufficient blood for lab testing.

In addition, anemia in preterm infants, especially in VLBWI, is a critical concern, as it leads to further complications such as respiratory distress, poor weight gain, and increased risk of infections. Anemia in these infants often requires blood transfusions, which carry additional risks, including infection, iron overload, and potential complications like retinopathy of prematurity (ROP) and necrotizing enterocolitis (NEC). If we can effectively reduce the occurrence or severity of iatrogenic anemia, decreasing the likelihood of blood transfusions in premature infants, it would be of great significance for the management of these infants. Whether umbilical cord blood (UCB) collection can reduce the risk of anemia and the chance of transfusion in VLBWI remain unclear (8, 9). Our study retrospectively collected clinical data to investigate the effects of UCB collection at birth on preterm infants with BW<1,500 g, and evaluated whether UCB collection can reduce blood sampling volume during hospitalization, indirectly decreasing the risk of anemia.

## 2 Methods

### 2.1 Study design

This study was a single-center retrospective observational study designed to evaluate the impact of UCB collection at birth on early clinical outcomes in preterm infants. Clinical data were retrospectively collected from hospital records of infants admitted to the NICU at a tertiary center in China, the Third Xiangya Hospital of Central South University from January 1, 2019 to August 31, 2023.

### 2.2 Patient population

Premature infants admitted to the NICU were eligible for inclusion if they met the following criteria: (1) gestational age (GA) < 37 weeks; (2) BW < 1,500 g; (3) availability of clinical data during hospitalization. Exclusion criteria included: (1) congenital malformations or known genetic/metabolic disorders; (2) transfer to another hospital, treatment withdrawal, or death during hospitalization; (3) acute massive bleeding or hemolysis at birth or in the early postnatal period. Although the inclusion criteria allowed gestational age up to 37 weeks, the actual study consisted of infants with a maximum gestational age of 31 weeks, reflecting the real-world NICU admission profile during the study period.

### 2.3 Group definition

Infants were divided into two groups based on whether umbilical UCB was collected: the UCB group and the NUCB group. The decision to collect UCB was based on physicians' habits and clinical conditions (e.g., difficulties in blood collection). UCB is collected through the umbilical vessels on the placental side after the umbilical cord is cut. UCB samples were primarily used for early laboratory testing, including complete blood counts, biochemical analyses, and microbiological investigations. Delayed cord clamping and cord milking were performed in accordance with *China neonatal resuscitation guideline (revised in 2021)* (10).

Analysis was conducted based on two subgroups according to body weight: the group with BW ≥ 1,250 g and the group with BW < 1,250 g to partially account for differences in physiological vulnerability among preterm infants.

### 2.4 Data collection

Demographic, perinatal, and clinical data were extracted from electronic medical records. Baseline variables included BW, GA, sex, length of hospital stay, respiratory support within 24 h after birth, and readmission within six months after discharge. Additional baseline factors, including maternal characteristics, antenatal steroid exposure, small-for-gestational-age (SGA) status, and need for resuscitation at birth, were collected and analyzed.

Clinical outcomes included the occurrence of hemoglobin (Hb) < 120 g/L within the first week of life, blood transfusion during hospitalization, timing of the first transfusion, volume of blood drawn during the first 24 h and first three days after birth, early weight change, and major neonatal complications, including bronchopulmonary dysplasia (BPD), NEC, ROP, intraventricular hemorrhage (IVH), periventricular leukomalacia (PVL), and pulmonary hemorrhage. Baseline Hb values at birth were not consistently available due to the retrospective nature of the study and therefore were not included in the primary analyses. Red blood cell transfusions were administered in accordance with *Practice in Neonatology* (11).

### 2.5 Ethical statement

The study was reviewed and approved by the Ethics Committee of the third Xiangya Hospital of Central south university (approval number: FAST-23673), in accordance with the Declaration of Helsinki and relevant ethical guidelines. Given the retrospective design of the study, the requirement for informed consent was waived by the Ethics Committee, and all data were anonymized to ensure the protection of participant confidentiality. The study protocol was retrospectively registered in the Chinese Clinical Trial Registry (ChiCTR2500113741).

### 2.6 Statistical analysis

Continuous variables are presented as $\left(\bar{x}\pm \;s\right)$ or M (P25, P75), as appropriate, and were compared using Student's *t*-test or the Mann–Whitney *U*-test. Categorical variables are presented as counts and percentages and were compared using the chi-square test. Given the retrospective design and limited sample size, no multivariable regression or propensity score-based adjustment was performed. Statistical analyses were performed using SPSS version 29.0, with a two-tailed significance level set at *P* < 0.05.

## 3 Results

### 3.1 General information

A total of 138 neonates met the inclusion criteria, including 68 infants in the UCB group and 70 infants in the NUCB group. Among them, 64 infants had BWs between 1,250 and 1,499 g and 74 infants had BWs <1,250 g.

There were no significant differences between the UCB group and NUCB group in maternal characteristics, including maternal age, antenatal steroid exposure, complete course of antenatal steroids, pregnancy-induced hypertension or preeclampsia, gestational diabetes mellitus, chorioamnionitis, multiple gestation, Intrauterine growth restriction (IUGR), or mode of delivery (*P* > 0.05, see Table 1).

**Table 1:** Maternal and neonatal clinical characteristics of premature infants with birth weight <1,500 g in the UCB and NUCB groups.

Variable	UCB (*n* = 68)	NUCB (*n* = 70)	*t/Z/χ^2^*	*P*
Maternal's condition				
Maternal age, [median (IQR), y]	29（25–33）	31（27–34）	−1.923^b^	.055
Antenatal steroid exposure (n,%)	59（86.8）	62（88.6）	0.104^b^	.747
Complete course of antenatal steroids (n,%)	48（70.6）	53（75.7）	0.462^b^	.497
Pregnancy-induced hypertension/preeclampsia (n,%)	16（23.5）	14（20.0）	0.253^b^	.615
Gestational diabetes mellitus (n,%)	11（16.2）	13（18.6）	0.138^b^	.710
Chorioamnionitis (n,%)	9（13.2）	7（10.0）	0.352^b^	.553
Multiple gestation (n,%)	21（30.9）	19（27.1）	0.234^b^	.628
Intrauterine growth restriction (n,%)	22（32.4）	20（28.6）	0.268^b^	.605
Cesarean delivery (n,%)	45（66.2）	48（68.6）	0.090^b^	.764
Neonatal's condition				
Birth weight [median (IQR), g]	1,183 (1,004, 1,288)	1290 (1168, 1410)	−3.644^b^	<.001
Gestational age (mean ± SD, w)	29.3 ± 2.8	30.1 ± 1.7	−2.073	.011
Small for gestational age (n,%)	21（30.9）	19（27.1）	0.247	.619
Length of hospital stay [median (IQR), d]	51.0 (42.9, 69.9)	44.3 (34.6, 60.3)	−2.284^b^	.022
Male sex (n,%)	31 (45.6）	39 (55.7)	1.415^a^	.234
Readmission rate within six months after birth (n, %)	5 (7.4)	5 (7.1)	0.002^a^	.962
Need for resuscitation at birth (n,%)	12（17.6）	13（18.6）	0.020^a^	.888
Apgar score at 1 min [median (IQR), min]	8（8–9）	8（6–9）	−1.203^b^	.229
Apgar score at 5 min [median (IQR), min]	10（9–10）	10（9–10）	−0.013	.990
Respiratory support at 24 h after birth (n, %)			5.522^a^	.137
Invasive	29 (42.6)	17 (24.3)		
Non-invasive	34 (50.0)	47 (67.1)		
High-Flow/Oxygen mix	1 (1.5)	2 (2.9)		
No respiratory support	4 (5.9)	4 (5.7)		

Superscript ‘^a^’ indicates the Chi-square (χ^2^) test; superscript ‘^b^’ indicates the Z value from the Mann–Whitney U test; values without superscripts indicate the independent- samples t test. Years(y), weeks(w), days(d), Umbilical Cord Blood Collection (UCB), Non-Umbilical Cord Blood Collection (NUCB). Values are presented as median (IQR), mean ± SD deviation, or *n* (%), as appropriate. Percentages are calculated based on the total number of participants in each group. All *p*-values are two-tailed, with statistical significance set at *p* < .05. Percentages are rounded to one decimal place.

Compared with the NUCB group, infants in the UCB group had a significantly lower BW and gestational age, as well as a significantly longer length of hospital stay (*P* < 0.05). No significant differences were observed between the two groups in sex distribution, small for gestational age (SGA), need for intubation at birth, Apgar scores at 1 and 5 min, respiratory support at 24 h after birth, or readmission rates within six months after birth (*P* > 0.05, see Table 1). No severe BPD (sBPD), ROP stage III or higher, or IVH (grade III-IV) occurred in either group.

### 3.2 Impact of UCB on preterm infants with BW < 1,250 g

Among preterm infants with BW < 1,250 g, the incidence of Hb <120 g/L within the first week after birth, as well as the amount of blood drawn within 24 h and the three days after post-birth, were significantly lower in the UCB group compared to the NUCB group (*P* < 0.05). Furthermore, the rate of non-transfusion within the first 7 days after birth was significantly higher in the UCB group (*P* < 0.05). (see Tables 2, 3).

**Table 2:** Analysis of the impact of umbilical cord blood collection on preterm infants of different birth weight categories (BW < 1,500 g*).*

Variable	BW < 1,250 g				BW ≥1250g			
	UCB (*n* = 47)	NUCB (*n* = 27)	*t/χ^2^*	*P*	UCB (*n* = 21)	NUCB (*n* = 43)	*t/χ^2^*	*P*
Occurrence of Hb <120 g/L within the first week of life (n, %)	14 (31.9)	15 (55.6)	4.778^a^	.029	8 (38.1)	12 (27.9)	0.682^a^	.409
Blood transfusions during hospitalization (n, %)	41 (87.2)	22 (81.5)	0.448^a^	.503	11 (52.4)	19 (44.2)	0.381^a^	.537
No Transfusion within 7 days after birth (n, %)	31 (66.0)	11 (40.7)	6.830^a^	.033	17 (80.9)	39 (90.7)	1.260^a^	.533
1-Week weight gain rate (mean ± SD, g/kg.d)	−4.7 ± 4.8	−4.4 ± 5.7	−0.243	.808	−2.1 ± 6.3	−2.9 (5.3)	0.517	.607
Blood drawn within 24 h after birth (mean ± SD,mL/kg)	5.5 ± 2.1	9.6 ± 3.1	−6.904	<.001	4.9 ± 2.1	7.2 ± 2.2	−3.961	<.001
Blood drawn within 3 days after birth (mean ± SD, mL/kg)	9.0 ± 3.0	13.8 ± 4.6	−5.486	<.001	7.1 ± 2.1	8.8 ± 2.3	−2.767	.007
Duration of respiratory support (mean ± SD, d)	51.0 ± 25.4	47.9 ± 22.8	0.531	.597	27.2 ± 18.1	27.0 ± 16.8	0.035	.972

Birth Weight (BW), Umbilical Cord Blood Collection (UCB), Non-Umbilical Cord Blood Collection (NUCB). Values are presented as mean (SD) for continuous variables and *n* (%) for categorical variables. Superscript ‘^a^’ represents the Chi-square (χ^2^) test, values without superscripts indicate the independent- samples t test. Weight gain rate=(Weight on the seventh day - Admission weight)/Admission weight. Blood drawn within 24 h=Total blood drawn within 24 h/Admission weight. Blood drawn within 3 days=Total blood drawn within 3 days/Admission weight. ‘p’ values are from two-tailed tests with significance set at *p* < .05. All percentages are rounded to one decimal place to provide precise statistical information while avoiding unnecessary complexity.

**Table 3:** Impact analysis of cord blood collection on complications in preterm neonates across different birth weight categories (BW < 1,500 g*).*

Complications (n, %)	BW < 1,250 g				BW ≥1250g			
	UCB (*n* = 47)	NUCB (*n* = 27)	χ^2^	*p*	UCB (*n* = 21)	NUCB (*n* = 43)	χ^2^	*p*
BPD	24 (51.1)	12 (44.4)	0.301	.583	2 (9.5)	9 (20.9)	1.290	.256
NEC	11 (23.4)	2 (7.4)	3.030	.082	2 (9.5)	3 (7.0)	0.127	.721
NEC (≧Ⅱb stage)	1 (2.1)	0 (0.0)	0.582	.445	1 (4.8)	0 (0.0)	2.080	.149
ROP	22 (46.8)	14 (51.9)	0.175	.676	5 (23.8)	7 (16.3)	0.525	.469
ROP II stage plus	4 (8.5)	3 (11.1)	0.135	.713	0 (0.0)	0 (0.0)	1.000	1.00
IVH-PVL	1 (2.1)	0 (0.0)	0.582	.445	0 (0.0)	0 (0.0)	1.000	1.00
IVH	8 (17.0)	2 (7.4)	1.356	.244	3 (14.3)	4 (9.3)	0.360	.549
PVL	0 (0.0)	0 (0.0)	1.000	1.00	1 (4.8)	0 (0.0)	2.080	.149
PH	3 (6.4)	3 (11.1)	0.515	.473	0 (0)	1 (2.3)	0.496	.481

Birth Weight (BW), Umbilical Cord Blood Collection (UCB), Non-Umbilical Cord Blood Collection (NUCB). Bronchopulmonary Dysplasia (BPD), Necrotizing Enterocolitis (NEC), Retinopathy of Prematurity (ROP), Intraventricular Hemorrhage with Periventricular Leukomalacia (IVH-PVL), Intraventricular Hemorrhage (IVH), Periventricular Leukomalacia (PVL), Pulmonary Hemorrhage (PH). Percentages represent the incidence of each complication within different birth weight categories (BW <1250 g, BW between 1,250–1,499 g). Values indicate the number of neonates with a specific complication and the percentage they represent [e.g., 24 (51.1%) indicates that 24 neonates have the condition, constituting 51.1% of the total neonates in that group]. When the percentage is 0 [e.g., 0 (0%)], it indicates no observed instances of the complication in that specific group. All percentages are rounded to one decimal place to provide precise statistical information while avoiding unnecessary complexity.

### 3.3 Impact of UCB on preterm infants with BW ≥ 1250g

In preterm infants with BW **≥** 1,250 g, the amount of blood drawn within 24 h and the first three days after birth was significantly lower in the UCB group than in the NUCB group (*P* < 0.05) (see Tables 2 and 3).

## 4 Discussion

Blood volume accounts for approximately 10% of a newborn's body weight (12). Preterm infants have a relatively small effective circulating blood volume because of their low body weight, and repeated blood draws for laboratory testing may result in substantial iatrogenic blood loss. Consequently, iatrogenic blood loss is considered an important contributor to anemia in preterm infants (13). When the amount of blood drawn reaches 4–8 mL/kg, it corresponds to approximately 5%–10% of the total circulating blood volume in preterm infants, which may lead to clinically significant hemodynamic changes and even life-threatening events (14). Severe anemia in very low BW infants represents an important clinical concern and is closely associated with an increased likelihood of red blood cell transfusion, particularly among infants requiring intensive respiratory support (15).

In this retrospective study, UCB collection at birth was associated with a significant reduction in early blood loss during the early postnatal period in preterm infants with BW <1,250 g. Specifically, infants in the UCB group required substantially lower amount of blood drawn within the first 24 h and the first three days after birth, which together constitute the most critical hematologic period during the first week of life. These findings indicate that UCB collection can effectively decrease early iatrogenic blood loss at a time when circulating blood volume is extremely limited (13).

Importantly, the clinical impact of this reduction appeared to be BW dependent. Among infants with BW < 1,250 g, UCB collection was not only associated with lower blood draw volumes but also with a significantly lower incidence of Hb <120 g/L within the first week of life and a higher proportion of infants who avoided transfusion during the first 7 days after birth. In contrast, although UCB collection reduced blood sampling volumes in infants with BW ≥ 1,250 g, no significant differences were observed in early anemia or transfusion outcomes. This suggests that infants with lower BW, who have narrower physiological reserves and are more vulnerable to even modest blood loss, may derive greater hematologic benefit from strategies that minimize early postnatal blood draw volume (16).

From a clinical perspective, UCB collection at birth may offer several advantages. First, it may reduce early postnatal iatrogenic blood loss by decreasing the amount of blood drawn after admission. Second, by reducing early blood draw volume, it may help lower the risk of early anemia and decrease the likelihood of transfusion exposure, particularly in infants with BW <1,250 g. Third, reducing the need for repeated blood draws may decrease cumulative blood loss, alleviate pain associated with blood draws procedures, and reduce the risk of iatrogenic anemia (17, 18). Together, these potential benefits support the incorporation of UCB collection as an early blood management strategy for preterm infants with BW < 1,500 g.

This study has several limitations. First, its retrospective, single-center design may introduce selection bias, and the decision to collect UCB was influenced by physicians' individual practice habits and clinical conditions rather than random allocation. Second, baseline hemoglobin values at birth were not consistently available and therefore could not be included in the analysis. Third, due to the relatively limited sample size, multivariable adjustment was not performed, and residual confounding cannot be excluded. Finally, this study focused on early postnatal outcomes, and longer-term hematologic and neurodevelopmental outcomes were not evaluated. Prospective, multicenter studies with standardized protocols are warranted to further validate these findings.

## 5 Conclusion

UCB collection can effectively reduce the amount of blood drawn from preterm infants, particularly those with BW <1,500 g. This reduction in early blood sampling may help decrease the risk of iatrogenic anemia and potentially reduce the need for blood transfusions. However, despite these potential benefits, the results should be interpreted with caution, particularly in broader clinical practice, as individual clinical factors and variations in transfusion practices may influence transfusion decisions.

## Data Availability

The raw data supporting the conclusions of this article will be made available by the authors, without undue reservation.
